# A Case Report of Calyceal Diverticulum: Differential Diagnosis for Organ-Preserving Operations

**DOI:** 10.3389/fsurg.2021.731796

**Published:** 2021-09-16

**Authors:** Alexandr V. Kurkov, Viktoriya M. Pominalnaya, Viktor V. Nechay, Igor A. Ratke, Sergej V. Mishugin, Alexandr A. Drobyazko, Alexandra V. Butenko, Alexey L. Fayzullin, Ekaterina A. Gomzikova

**Affiliations:** ^1^Department of Anatomic Pathology, D.D. Pletnev City Clinical Hospital, Moscow Health Department, Moscow, Russia; ^2^Institute for Regenerative Medicine, Sechenov First Moscow State Medical University (Sechenov University), Moscow, Russia; ^3^Department of Anatomic Pathology, City Clinical Hospital No 29 Named After N.E. Bauman, Moscow, Russia; ^4^Department of Oncourology, D.D. Pletnev City Clinical Hospital, Moscow Health Department, Moscow, Russia; ^5^Department of Anatomical Pathology, Sechenov First Moscow State Medical University (Sechenov University), Moscow, Russia; ^6^World-Class Research Center “Digital Biodesign and Personalized Healthcare”, Sechenov First Moscow State Medical University (Sechenov University), Moscow, Russia

**Keywords:** calyceal diverticulum, intrarenal epidermal cysts, simple renal cyst, kidney, nephrectomy

## Abstract

Calyceal diverticula and epidermal cysts are extremely rare kidney lesions with unknown etiology and pathogenesis. They have non-specific clinical and radiological picture. Despite the benign nature, sometimes these disorders mimic malignant tumors leading to unjustified nephrectomy. We present a clinical and morphological observation of a multicystic lesion in a 76-year-old patient's right kidney filled with keratinized masses and imitating a malignant solid tumor. The detailed gross, histological and immunohistochemical (desmin, cytokeratin 7, uroplakin and p63) analyses of the kidney tissue excluded the malignant nature of the lesion. The final differential diagnosis was between an epidermal cyst and calyceal diverticulum with pronounced squamous cell metaplasia of urothelium. The upper pole localization of the lesion, its connection with the pelvicalyceal system through the unobstructed isthmus, the presence of urothelial lining and smooth muscle cells in its wall let us diagnose a calyceal diverticulum type I. Knowledge of the key clinical and morphological features of epidermal cysts and diverticula of the pelvicalyceal system will help the practicing physicians suspect the benign nature of such lesions and perform organ-preserving operations.

## Introduction

Benign kidney lesions, such as calyceal diverticulum and intrarenal epidermal cyst, are rare and do not have specific clinical symptoms (1–7). However, they can be mistaken as kidney cancers leading to unjustified nephrectomy (2, 4, 5, 7).

Calyceal diverticula are outpouchings from the calyx or pelvis with epithelial lining, muscular layer and narrow channel connecting the diverticulum cavity with the central collecting system (1, 2, 8). Their size ranges from 0.5 to 7.5 cm (average value is 1.72 cm) sometimes reaching 18 cm (1). Calyceal diverticula do not have gender or age predisposition and can be detected accidentally in 0.21–0.6% of adults and children performing intravenous pyelographic investigation (1, 2). Diverticula are divided into two types: type I (calyceal diverticula) are more common, they communicate with a minor calyx and occur in the upper pole of the right kidney; type II (so called pyelocalyceal diverticula) communicate with a major calyx or the renal pelvis at the interpolar region of the kidney and tend to be larger (1, 2).

Epidermoid cyst of the kidney is an extremely rare pathology, only a few cases have been reported (3–7, 9–12). They are usually localized in the renal parenchyma and in the pelvicalyceal system. Their cavities are lined with the stratified squamous epithelium forming lamellar keratinized masses inside the lumen. Sometimes, the epithelium contains a developed granular layer and stratum corneum may be calcified.

The literature data shows that diverticula of the pelvicalyceal system and epidermoid cysts can be congenital pathologies occurring during the embryonic period (epidermal remnant of Wolffian duct, aberrant ectodermal implantation) or results of infection, vesicoureteral reflux, mechanical compression by the tumor, renal cyst rupture, urolithiasis causing squamous metaplasia, traumatic implantation or traumatic metaplasia because of surgical interventions (1, 2, 4, 9). Pyelocalyceal diverticula and epidermal cysts have a non-specific clinical picture and require differential diagnosis with various benign and malignant neoplasms of the kidney (2, 4, 7, 9). They can manifest with such symptoms as flank or loin pain, renal colic, urinary frequency, hematuria, infection or be asymptomatic. In addition, the diverticula contribute to nephrolithiasis, infection, hematuria and kidney failure, cause the compression of the surrounding tissues and facilitating the growth of malignant tumors (13–15).

At radiological examination, the diverticula of the pelvicalyceal system look like thin-walled cavities that communicate with the central collecting system. They can be empty or filled with urine, stones or milk of calcium (2). Epidermal cysts localized intrarenal or in the renal pelvis can form cavities filled with inhomogeneous calcified masses (4, 7). Sometimes these radiological features can mimic a malignant neoplasm leading to nephrectomy (5). In this case, the final diagnosis is verified only after the surgical removal of the kidney. It was reported (5, 16), that the biopsy of the lesion may suggest its benign nature requiring an organ-preserving surgery. However, this diagnostic tactic has not been approved yet.

Thus, this report will help radiologists, pathologists and urologists to learn about these rare conditions. Here, we demonstrate the clinical case of multicameral lesion of the upper pole of the right kidney in a 76-year-old patient who underwent surgery in 2018 due to suspected kidney cancer.

## Case Report

**Figure 1:**
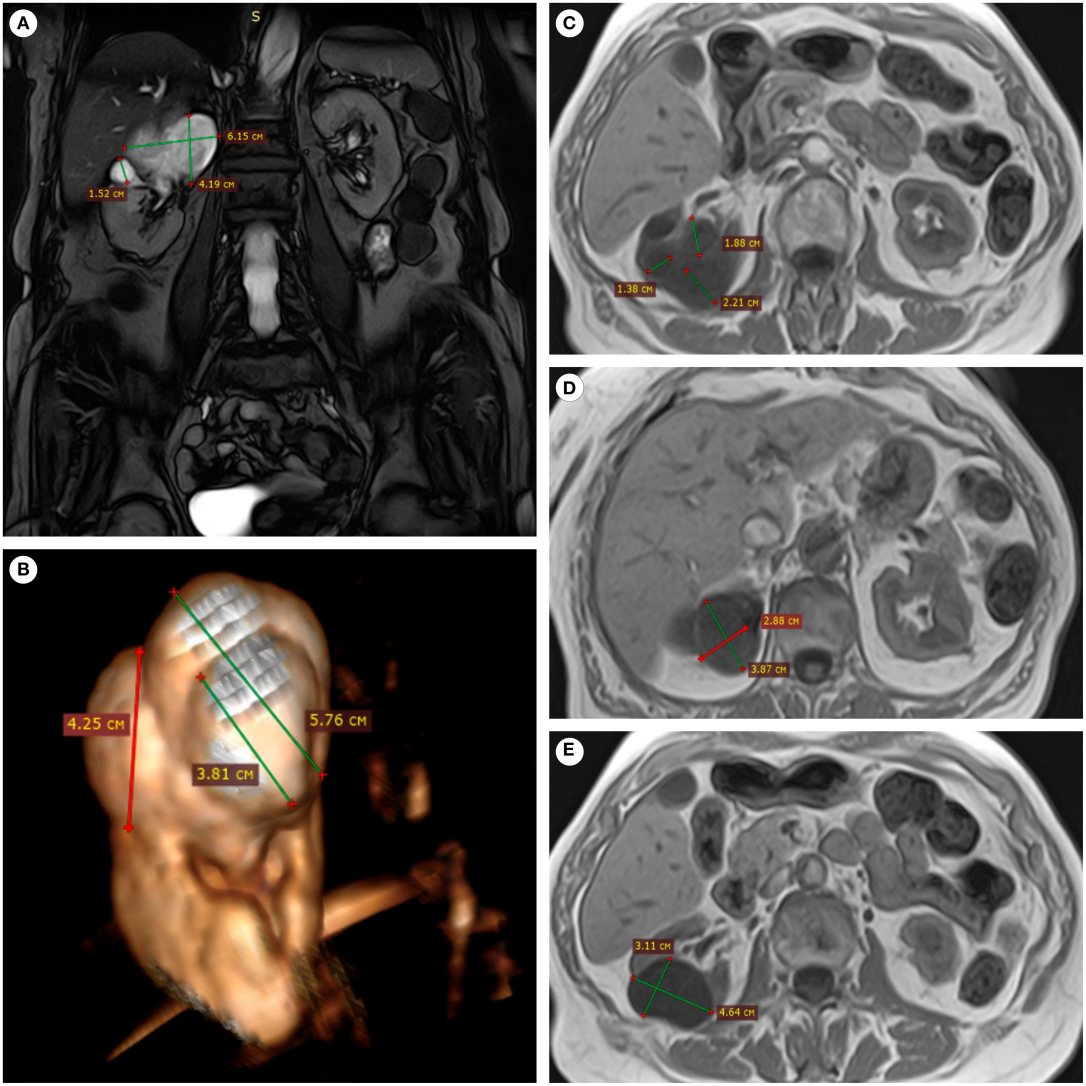
MRI of the patient's abdominal cavity (without contrast). **(A)** In the upper pole of the right kidney, the lesion of an irregular shape and inhomogeneous structure was connected with the pelvicalyceal system, coronal projection, SSFP mode. **(B)** The lesion had an irregular shape and a bumpy surface, 3D-reconstruction from the SSFP series. **(C–E)** The multinodular nature of the lesion and clear boundaries were determined in axial sections, T1 mode.

A 76-years-old woman came to the 2nd oncology department of D.D. Pletnev City Clinical Hospital with a suspected malignant neoplasm of the kidney (ICD-10:C64) in October 2018. Written informed consent was obtained from the individual for the publication of any potentially identifiable images or data included in this article. According to the anamnesis, she complained of pain in the right side for a long time. An outpatient ultrasound examination revealed a solid tumor-like structure in the upper pole of the right kidney 7 x 6 x 5 cm in size. In computer tomography (CT) plain scan and magnetic resonance imaging (Figure 1), the formation had irregular contours. Based on the radiology, the lesion had RENAL Nephrometry Score 10 meaning high complexity of the surgical intervention. It was also known that the patient suffered from hypertension, a permanent atrial fibrillation, cerebral atherosclerosis (stenosis of the internal carotid arteries up to 50%), chronic heart failure, postmenopausal duration−26 years. The patient's creatinine and urea levels at the time of hospitalization were within normal limits. The hospital surgeons carried out a planned laparoscopic right-sided nephrectomy supported by the clinical picture and radiology (Table 1).

**Table 1:** A table summarizing care checklist organized into a timeline.

**The event**	**Timeline**
Manifestation of pain in the right side	May 2018
MRI visualization of the kidney lesion	August 2018
Planned nephrectomy	October 2018

**Figure 2:**
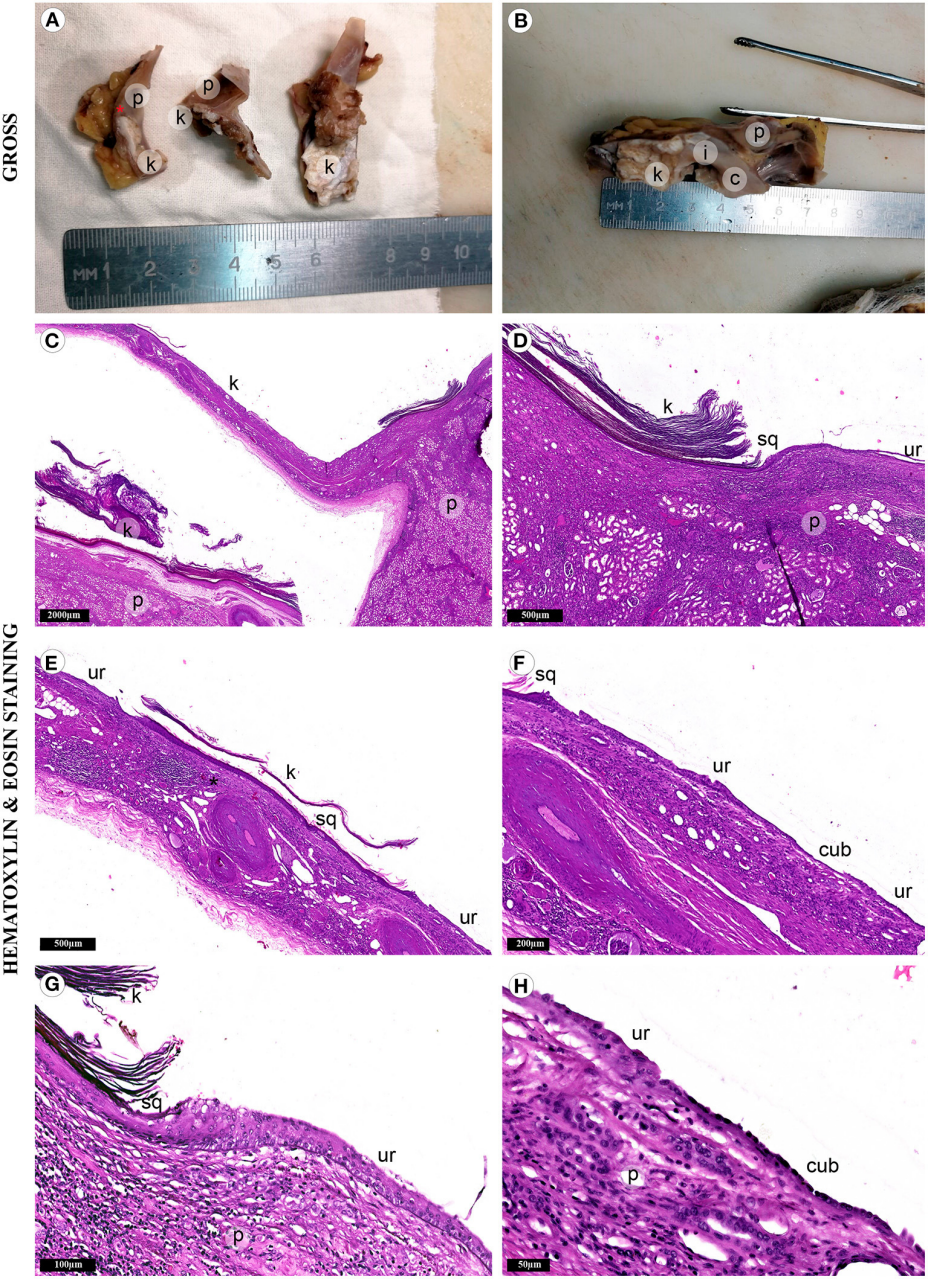
Fragments of the kidney and cystic lesion. Gross evaluation **(A,B)** demonstrates cystic lesion with adjacent renal parenchyma (p) separated by septa with varying wall thickness. Adhesions tightly connected the lesion with the kidney capsule and adjacent adipose tissue. The lumen was filled with gray-brown cheesy keratinized masses (k). It was connected to the small renal calyx (c) with the isthmus (i). Mucosa of the calyx was gray, smooth, with single overlays of horny masses. **(C)** The histological study revealed keratinized mass in the lumen of the lesion. **(D)** The renal parenchyma was separated from the cyst wall with adipose tissue of the renal sinus. **(E,F)** Three types of epithelium lined the cyst wall: stratified squamous epithelium (sq) with a developed granular layer and hyperkeratosis, urothelium (ur) and simple cuboidal epithelium (cub). **(G)** We also detected the focuses of chronic inflammatory infiltration. **(H)** Atrophic renal parenchyma was adjacent to the epithelial lining in some areas.

**Figure 3:**
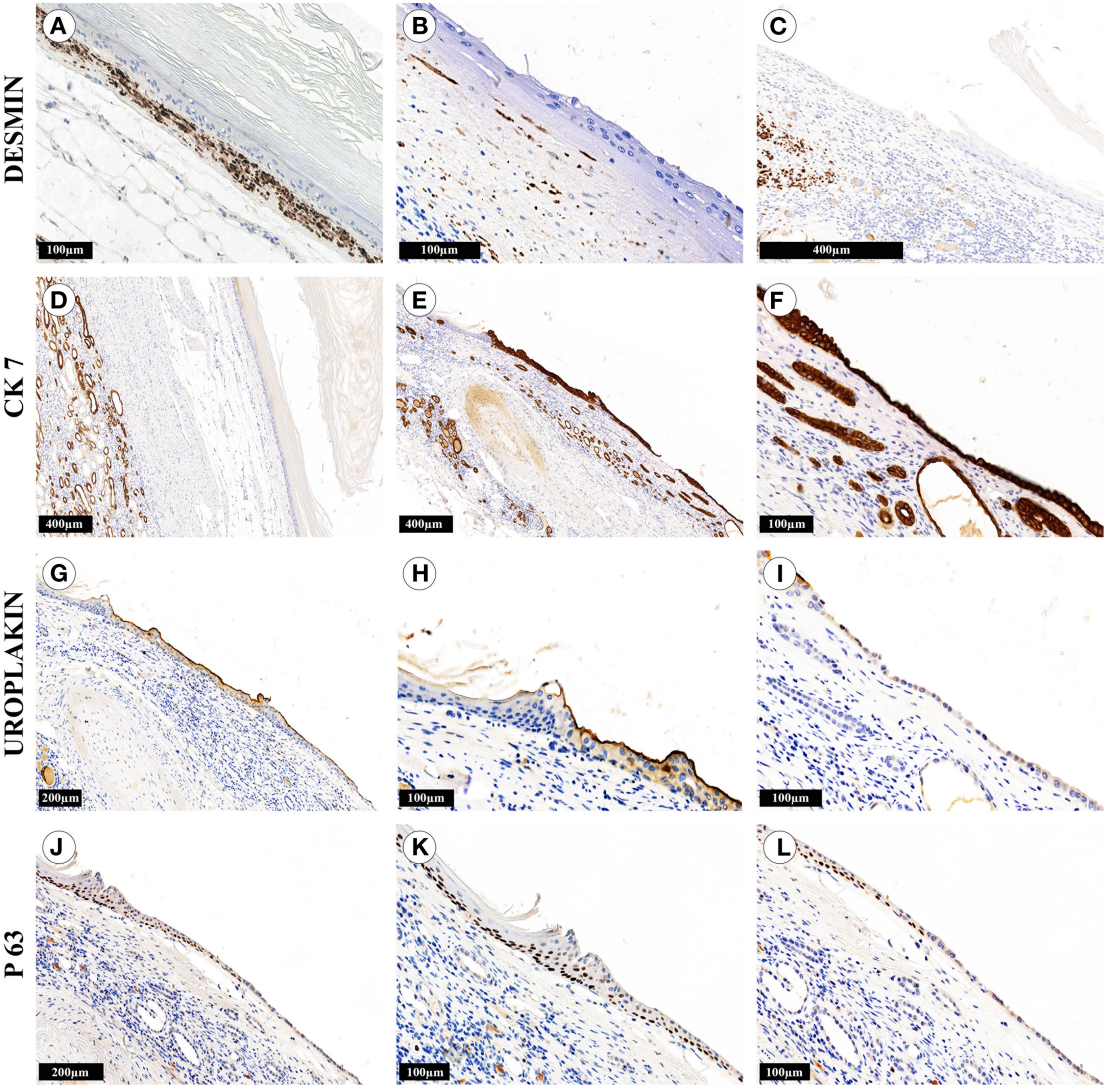
Immunohistochemical study of kidney cystic lesion. The cyst wall areas contained numerous smooth muscle cells **(A)**, singular cells **(B)** or completely lacked the cells **(C)**. **(D–F)** The cuboidal epithelium, urothelium and epithelium of the kidney tubules were strongly positive for CK7, while the stratified squamous epithelium did not express the marker. **(G–I)** The urothelium was positive for uroplakin III, while the cuboidal epithelium expressed this marker weakly and irregularly. **(J–L)** The multilayer squamous epithelium and tubule epithelium were not stained with antibodies against uroplakin III, but expressed p63. The cuboidal cyst epithelium had weak and focal positive expression of p63, the epithelium of the tubules was not stained.

Gross evaluation of the surgical material revealed a multichamber keratin-filled lesion in the upper pole of the right kidney, 7 x 6 x 5 cm in size (Figures 2A,B). The canal (isthmus) with a diameter of 0.7 cm connected it with one of the minor calices. The kidney parenchyma was thinned to 0.1 cm above this formation, while in other areas it was around 2 cm. In histological analysis (Figures 2C–H), the lining of the lesion consisted of alternating sections of urothelium and epidermis with developed granular and stratum corneum. The wall consisted of fibrous tissue with focuses of adipose cells, multi-caliber blood vessels and clusters of smooth muscle cells, which were clearly visualized by immunohistochemistry (Figures 3A–C). Urothelium and cuboidal epithelium of the cyst expressed cytokeratin 7 (CK7) and uroplakin 3, while the stratified squamous epithelium did not express these markers (Figures 3D–I). Both urothelium and squamous epithelium expressed p63 (Figures 3J–L).

In some areas, the wall of the lesion had well-defined contours and was separated from the renal parenchyma by renal sinus adipose tissue (Figure 2D and Figures 3A,D). We observed the areas with atrophy of the smooth muscle layer (Figure 3B). Somewhere, it was completely absent, the epithelial lining closely adjoined to the renal parenchyma and a single-layer cuboidal epithelium lined the wall (Figures 2F,H, and Figures 3C,E,F,I,L). Also, we defined the signs of chronic pyelonephritis with dilatation and deformation of the pelvicalyceal system. It is important to highlight, that we did not observe the appendages of the skin (hair, sebaceous and sweat glands) or any other components of teratoma-group lesions.

The patient had no complains 2.5 years after the nephrectomy. Blood and urine test results have remained normal.

## Discussion

The data of gross and microscopic studies excluded the malignant nature of the kidney lesion, as well as other pathologies that it could imitate (first of all, teratoma-group lesions) (1, 9). We observed extensive foci of squamous cell metaplasia without signs of cancer growth, in contrast to the previously reported cases (13–15).

To the best of our knowledge, this is the first report of differential diagnostics between the epidermal cyst of the kidney and the calyceal diverticulum with pronounced squamous cell metaplasia. The upper pole localization of the lesion, its connection with the pelvicalyceal system through the unobstructed isthmus, the presence of urothelial lining and smooth muscle cells in its wall let us diagnose a calyceal diverticulum type I. We suppose that squamous cell metaplasia of the urothelium was caused by traumatizing factors (in the present case, chronic pyelonephritis) (8). As a result, the diverticulum filled with keratinized masses imitated renal cell cancer in the radiological study that led to nephrectomy (1, 2).

Some areas of the multichamber lesion did not have muscle layer. It was lined with cuboidal epithelium and resembled the simple kidney cyst. It was hypothesized that the rupture of the simple kidney cyst (1) connected with diverticulum can result in squamous cell metaplasia of cyst lining and formation of multiple cavities.

Differential diagnosis between calyceal diverticula and epidermoid cysts is necessary for correct diagnosis and possible organ-preserving treatment, statistical accounting of these rare disorders, their complications and outcomes. Without better understanding of their pathogenesis and morphology, we lack criteria for definitive diagnosis, especially in cases of epidermal cysts that can actually be calyceal diverticula with squamous cell metaplasia (4, 5, 11, 12). However, besides standard methods of evaluation, immunohistochemistry can be useful to identify whether the lesion is a diverticulum or an epidermal cyst in complicated cases (for example, with severe atrophy of the smooth muscle layer in the wall of the diverticulum). Here, we investigated the key immunomorphological features of these two conditions on the nephrectomy material. Nevertheless, it is necessary to take into account the difficulties of diagnosing these lesions in biopsy material (5, 16).

There are currently no clinical or pathological guidelines devoted to the differential diagnosis of diverticula of the pelvicalyceal system with pronounced keratinization of the mucous membrane and epidermal cysts due to a small number of such observations and the absence of clear morphological criteria. In contrast to epidermal cysts, the identification of diverticula of the pelvicalyceal system does not require a mandatory morphological study, since methods of radiological diagnosis or percutaneous aspiration of the contents of the diverticulum cavity make it possible to make a diagnosis with great accuracy (2).

In our case, due to the presence of concomitant pathologies in the patient, it was impossible to carry out additional advanced diagnostics. The size and localization of the lesion did not allow to perform organ-preserving surgery. In this regard, a preliminary biopsy of the lesion was inappropriate and the final diagnosis was obtained only after a thorough histological examination of the removed kidney. We believe that the differential diagnosis between the diverticulum and renal cell cancer should be conducted at the preoperative stage when:

1. there is a possibility of organ-preserving surgery (absence of severe concomitant pathologies, low RENAL Nephrometry Score);
2. lesion is located in the upper pole and/or is associated with the pelvicalyceal system;
3. access to additional research methods (delayed intravenous pyelography, retrograde pyelography, percutaneous aspiration, biopsy);
4. accumulations of horny masses and/or elements of cystic lesion lined with multilayer squamous epithelium in the biopsy.

It is imperative to exclude a diverticulum of the pelvicalyceal system in cases where the patient has been diagnosed with an epidermal cyst of the kidney, especially when the cyst is located in the upper pole of the kidney or it has an anatomical connection with the renal calyx or pelvis. Pathologists should pay attention to the presence of smooth muscle cells in the cyst wall, since they may be the remnants of the muscle membrane of the renal calyx or pelvis. The presence of urothelium is not a mandatory sign of a diverticulum, since it can undergo various types of metaplasia (8). Only in the absence of an anatomical connection of the cyst with the pelvicalyceal system and smooth muscle cells in its wall can this lesion be reliably considered an epidermal cyst. Nevertheless, physicians should be aware of the risks of malignancy of diverticula and epidermal cysts.

All in all, diverticula of the pelvicalyceal system and epidermal cysts of the kidney are rare disorders which are difficult to diagnose. Knowledge of their key features will help to prevent unjustified nephrectomies, to optimize the statistics and to establish clinical guidelines for diagnosis and treatment of these diseases. We believe that this report will be useful for doctors of various specialties, especially for radiologists, pathologists and oncourologists.

## Data Availability Statement

The raw data supporting the conclusions of this article will be made available by the authors, without undue reservation.

## Ethics Statement

Written informed consent was obtained from the individual for the publication of any potentially identifiable images or data included in this article.

## Author Contributions

All authors listed have made a substantial, direct and intellectual contribution to the work, and approved it for publication.

## Funding

This work was financed by the Ministry of Science and Higher Education of the Russian Federation within the framework of state support for the creation and development of World-Class Research Centers Digital biodesign and personalized healthcare No. 075-15-2020-926.

## Conflict of Interest

The authors declare that the research was conducted in the absence of any commercial or financial relationships that could be construed as a potential conflict of interest.

## Publisher's Note

All claims expressed in this article are solely those of the authors and do not necessarily represent those of their affiliated organizations, or those of the publisher, the editors and the reviewers. Any product that may be evaluated in this article, or claim that may be made by its manufacturer, is not guaranteed or endorsed by the publisher.
